# Nomogram for predicting advanced liver fibrosis and cirrhosis in patients with chronic liver disease

**DOI:** 10.1186/s12876-021-01774-w

**Published:** 2021-04-27

**Authors:** Rongrong Ding, Xinlan Zhou, Dan Huang, Yanbing Wang, Xiufen Li, Li Yan, Wei Lu, Zongguo Yang, Zhanqing Zhang

**Affiliations:** 1 Department of Hepatobiliary Medicine, Shanghai Public Health Clinical Center, Fudan University, 2901 Caolang Road, Shanghai, 201508 China; 2 Department of Integrative Medicine, Shanghai Public Health Clinical Center, Fudan University, 2901 Caolang Road, Shanghai, 201508 China

**Keywords:** Nomogram, INR, Platelet, Liver fibrosis, Chronic liver disease

## Abstract

**Background:**

We aimed to formulate a novel predictive nomogram to discriminate liver fibrosis stage in patients with chronic liver disease.

**Methods:**

Nomograms were established based on the results of multivariate analysis. The predictive accuracy of the nomograms was assessed by ROC analysis and calibration. Decision curve analysis (DCA) was used to determine the clinical benefit of the nomograms.

**Results:**

INR, platelets, and N-terminal propeptide type III collagen (PIIINP) were independent predictors for advanced liver fibrosis (≥ S3) and cirrhosis (S4) in patients with chronic liver disease in the training cohort. In the training set, the areas under the ROCs (AUROCs) of nomogram S3S4, APRI, FIB-4, and GPR for stage ≥ S3 were 0.83, 0.71, 0.68, and 0.74, respectively; the AUROCs of nomogram S4, APRI, FIB-4, and GPR for stage S4 were 0.88, 0.74, 0.78, and 0.79, respectively. The calibrations showed optimal agreement between the prediction by the established nomograms and actual observation. In the validation set, the AUROCs of nomogram S3S4, APRI, FIB-4, and GPR for stage ≥ S3 were 0.86, 0.79, 0.78, and 0.81, respectively; the AUROCs of nomogram S4, APRI, FIB-4, and GPR for stage S4 were 0.88, 0.77, 0.81, and 0.83, respectively. Furthermore, the decision curve analysis suggested that the nomograms represent better clinical benefits in both independent cohorts than APRI, FIB-4, and GPR.

**Conclusion:**

The constructed nomograms could be a superior tool for discriminating advanced fibrosis and cirrhosis in chronic liver disease.

## Background

Chronic liver disease is a major public health problem that accounts for high morbidity and mortality worldwide. Their prognosis and treatment depend largely on the progression of liver fibrosis. The timely and accurate assessment of liver fibrosis is essential to prevent chronic liver disease from developing into cirrhosis, hepatic failure, and hepatocellular carcinoma [1]. To date, liver biopsy is still considered the gold standard for the assessment of liver fibrosis. Nevertheless, liver biopsy is an invasive procedure with some limitations, such as potentially life-threatening complications, high cost, observer discrepancies, and sampling errors [1]. Radiological methods including conventional ultrasound, computed tomography and magnetic resonance imaging can diagnose typical liver cirrhosis but cannot evaluate the presence or degree of liver fibrosis.

Therefore, many efforts to develop noninvasive methodologies that reflect the total amount of liver fibrosis have been made. Blood markers such as platelets and hyaluronic acid have been reported to be related to liver fibrosis [2, 3]. Serological markers such as the aspartate transaminase to platelet ratio (APRI), fibrosis-4 score (FIB-4), and γ-glutamyl transpeptidase to platelet ratio (GPR) have been used to assess liver fibrosis and have been reported to be effective [4–6]. However, they are controversial with respect to the evaluation of liver fibrosis stages [7–9]. Transient elastography has been developed as a procedure able to assess hepatic stiffness noninvasively [10, 11]. However, some factors, such as the need for expensive equipment and trained operators, may limit its clinical application [12]. Additionally, other potential mingle factors may also affect the accuracy of TE, such as obesity, increased transaminase, and operator skills [13].

A nomogram is an effective model for visualizing regression equations because it can establish scoring standards based on the regression coefficients of all independent variables [14]. Compared with other prediction models, prediction based on nomograms is more accurate. This method has been proposed as an alternative method or even a new standard method and has been widely used as a prognostic tool for many tumor types, such as hepatocellular carcinoma, lung cancer, renal cell carcinoma, and colorectal cancer [15–19].

The aim of our study was to explore the predictive significance of serological indexes such as serum fibrosis markers, coagulation factors, aminotransferase, bilirubin, and platelets for staging liver fibrosis. Furthermore, we wished to establish a predictive nomogram based on fibrosis indicators with prognostic significance. In addition, a comparison was made between the nomogram and other noninvasive models, including ARPI, FIB-4, and GPR.

## Methods

### Patients

Overall, between January 2016 and September 2020, we retrospectively recruited 701 consecutive Chinese individuals with chronic liver disease. The enrolled patients were divided into two time groups; the first 427 patients admitted to our hospital between January 2016 and December 2019 formed the training set, while the remaining 274 patients admitted between January 2020 and September 2020 formed the validation set. All patients underwent a careful clinical examination and liver biopsy at Shanghai Public Health Clinical Center, Fudan University. The inclusion criteria were diagnosed patients with treatment-naïve chronic viral hepatitis (hepatitis B or C), alcoholic liver disease, nonalcoholic fatty liver, and autoimmune hepatitis. All the patients were > 18 years old. The exclusion criteria were hepatocellular carcinoma, antiviral treatment history, decompensated cirrhosis, inadequate liver biopsy samples (< 1.5 cm), and the use of anticoagulant drugs.

### Liver biopsy

Percutaneous liver biopsy was performed using a 16 G needle under ultrasound guidance. Liver samples with a minimum length of 1.5 cm and at least 7 complete portal tracts were fixed in 10% formalin, embedded in paraffin, and stained with HE, Masson’s trichrome and reticulin staining for histological analysis [20]. Liver histology was analyzed by two experienced pathologists who were blinded to other clinical and laboratory data and classified according to the Scheuer scoring system [21] as follows: S0 (no fibrosis), S1 (mild fibrosis without septa), S2 (moderate fibrosis with few septa), S3 (severe fibrosis with numerous septa without cirrhosis), and S4 (cirrhosis). In this study, liver fibrosis stage S0-S2 was defined as mild/moderate fibrosis, and S3-S4 was defined as advanced fibrosis.

### Laboratory data

Fasting blood samples were obtained within a week of liver biopsy. Platelets and other blood cells were counted using a Sysmex-XT 4000i automated hematology analyzer. The international normalized ratio (INR) and other coagulation indexes were measured using a STAR Max automatic coagulation analyzer. Alanine transaminase (ALT), aspartate aminotransferase (AST), alkaline phosphatase (ALP), γ-glutamyl transferase (GGT), hyaluronic acid, laminin, N-terminal propeptide of type III procollagen (PIIINP), type IV collagen, and other serum biochemical parameters were measured using an Architect C16000 automatic biochemical analysis system.

### Formulas

The formulas for APRI, FIB-4, and GPR are as follows: APRI = (AST (U/L)/ULN of AST)/platelet count (10^9^/L) × 100; FIB-4 = (age (years) × AST (U/L))/(platelet count (10^9^/L) × (ALT (U/L))^1/2^); GPR = (GGT (U/L)/ULN of GGT)/platelet count (10^9^/L) × 100.

### Statistical analysis

Statistical analysis was performed using IBM SPSS Statistics version 26.0 (SPSS Inc., Chicago, USA) and R 4.0.2 (http://www.R-project.org). Continuous variables are expressed as the mean ± standard deviation or median (interquartile range, IQR) and compared using the Student’s *t*-test (for nomal distribution continuous variables) or independent Mann–Whitney *U*-test (for non-nomal distribution continuous variables). Categorical variables are expressed as proportions and compared by the chi-square test. Logistic regression models were used to assess the correlation between variables and liver fibrosis. A nomogram for evaluating liver fibrosis was established based on the results of the multivariate analysis and by using the rms package in R. The predictive accuracy of the nomogram was assessed by calibration. The performances of the established nomogram and other noninvasive markers for predicting liver fibrosis were assessed by receiver operating characteristic (ROC) curve analyses. Delong Z test was used to compare the AUROC of the serum models. Decision curve analysis (DCA) was used to further evaluate the predictive performances. A two-sided P < 0.05 was considered statistically significant.

## Results

### Baseline demographic and clinical characteristics

The demographic and clinical characteristics of the studied patients with chronic liver disease are described in Table 1. Overall, the majority of patients suffered from chronic hepatitis B, and there were no significant differences in various clinical parameters between the training set and validation set. Of the training set, 238 patients had biopsy-proven fibrosis S0-S2, and 189 patients had fibrosis S3-S4. ALT, AST, ALP, GGT, DBil, globulin, total bile acid, INR, prothrombin time, APTT, fibrinogen, and thrombin time levels were significantly higher in subjects with fibrosis stages S3-S4 than in subjects with fibrosis stages S0-S2. The levels of albumin, prealbumin, platelet counts, and neutrophil counts were significantly lower in patients with stages S3-S4. In addition, compared with patients with stages S0-S2, the values of biochemical scores significantly increased in patients with stage S3-S4.

**Table 1:** Clinical characteristics of studied patients with chronic liver disease

Variables	Total (n = 701)			Training cohort (n = 427)		
	Training set (n = 427)	Validation set (n = 274)	*P* value	S0–S2 (n = 238)	S3–S4 (n = 189)	*P* value
Age, years	37 (31–46)	36 (30–46)	0.171	38 (31–46)	37 (31–45)	0.488
Male, n (%)	284 (66.5)	181 (66.1)	0.902	158 (66.4)	129 (68.3)	0.683
Aetiology						
Chronic hepatitis B, n (%)	263 (61.6)	220 (80.3)	< 0.001	135 (56.8)	128 (67.7)	0.020
Chronic hepatitis C, n (%)	40 (9.4)	8 (3.0)	0.001	20 (8.4)	20 (10.7)	0.552
NAFLD, n (%)	43 (10.1)	30 (10.9)	0.710	31 (13.0)	12 (6.3)	0.023
Alcoholic liver disease, n (%)	30 (7.0)	5 (1.8)	0.002	25 (10.5)	5 (2.6)	0.002
Autoimmune hepatitis, n (%)	51 (11.9)	11 (4.0)	< 0.001	27 (11.3)	24 (12.7)	0.668
Blood parameters						
ALT, U/L	56.00 (31.00–123.00)	67.00 (28.00–148.50)	0.320	50.50 (23.00–112.50)	66.00 (38.00–143.50)	0.002
AST, U/L	41.00 (26.00–76.00)	41.00 (24.00–96.25)	0.378	33.50 (22.00–62.25)	50.00 (31.00–89.50)	< 0.001
ALP, U/L	77.00 (64.00–93.00)	75.00 (63.00–99.00)	0.574	72.00 (59.75–86.25)	85.00 (67.00–101.50)	< 0.001
GGT, U/L	35.00 (19.00–67.00)	35.00 (19.00–86.00)	0.500	26.00 (16.00–47.00)	49.00 (28.50–85.00)	< 0.001
TBil, μmol/L	15.05 (11.10–19.85)	14.60 (10.08–20.28)	0.800	14.40 (11.15–18.85)	15.70 (11.10–21.70)	0.054
DBil μmol/L	5.50 (4.10–7.50)	5.25 (3.70–8.13)	0.414	5.25 (4.00–6.80)	6.10 (4.60–8.70)	< 0.001
Albumin, g/L	42.00 (39.20–44.50)	42.20 (39.88–44.53)	0.403	42.80 (40.20–45.25)	41.00 (38.20–43.75)	< 0.001
Globulin, g/L	29.00 (26.00–32.00)	27.00 (24.00–30.00)	< 0.001	29.00 (26.00–32.00)	30.00 (26.50–33.00)	0.034
Prealbumin, g/L	200.20 (151.00–249.35)	209.00 (145.00–258.00)	0.389	217.00 (170.25–269.00)	170.00 (127.50–218.00)	< 0.001
Total bile acid, μmol/L	8.75 (4.20–15.60)	9.70 (5.50–20.75)	0.003	6.70 (3.50–12.00)	11.80 (6.60–20.60)	< 0.001
FBG, mmol/L,	4.79 (4.49–5.21)	4.90 (4.57–5.32)	0.060	4.82 (4.57–5.24)	4.74 (4.46–5.15)	0.032
TC, mmol/L	4.14 (3.66–4.83)	4.07 (3.57–4.67)	0.106	4.27 (3.79–4.89)	3.98 (3.50–4.68)	0.001
TG, mmol/L	0.93 (0.71–1.24)	1.00 (0.75–1.40)	0.022	0.96 (0.75–1.31)	0.88 (0.70–1.11)	0.006
HDL, mmol/L	1.32 (1.06–1.64)	1.32 (1.06–1.54)	0.278	1.34 (1.04–1.63)	1.31 (1.08–1.67)	0.930
LDL mmol/L	2.60 (2.11–3.18)	2.45 (2.02–3.07)	0.017	2.70 (2.27–3.32)	2.40 (2.01–2.91)	< 0.001
Urea, mmol/L	314.00 (257.00–370.18)			314.80 (248.05–381.50)	312.55 (268.38–356.08)	0.682
Creatinine, μmol/L	65.25 (55.17–75.10)	68.10 (56.30–77.20)	0.124	63.75 (55.50–74.50)	67.15 (54.50–75.73)	0.330
Prothrombin time, s	13.80 (13.20–14.40)	13.60 (13.00–14.30)	0.021	13.50 (13.10–14.00)	14.10 (13.50–15.00)	< 0.001
INR	1.05 (1.00–1.12)	1.05 (0.99–1.10)	0.075	1.03 (0.99–1.08)	1.09 (1.03–1.17)	< 0.001
APTT, s	38.60 (36.30–41.20)	38.65 (35.83–41.78)	0.877	37.70 (35.60–40.00)	39.80 (37.30–42.20)	< 0.001
Fibrinogen, g/L	2.50 (2.17–2.79)	2.38 (2.04–2.64)	0.001	2.59 (2.25–2.90)	2.36 (2.09–2.62)	< 0.001
Thrombin time, s	17.70 (17.00–18.50)	17.80 (17.00–18.70)	0.508	17.45 (16.90–18.23)	18.10 (17.30–18.80)	< 0.001
WBC count, × 10^9^/L	5.28 (4.23–6.27)	5.24 (4.33–6.25)	0.595	5.41 (4.39–6.45)	5.14 (4.12–6.14)	0.057
RBC count, × 10^9^/L	4.65 (4.26–5.01)	4.56 (4.12–4.93)	0.004	4.75 (4.35–5.06)	4.54 (4.12–4.95)	0.003
Platelet count, × 10^9^/L	159.00 (130.00–195.00)	152.50 (122.00–185.25)	0.066	177.00 (150.00–207.25)	140.00 (98.50–171.50)	< 0.001
Hemoglobin (g/L)	145.00 (132.00–156.00)	141.00 (128.00–154.00)	0.014	147.00 (133.75–157.00)	143.00 (129.00–155.00)	0.118
Neutrophils count, × 10^9^/L	2.84 (2.18–3.58)	2.69 (2.07–3.42)	0.083	2.98 (2.35–3.65)	2.62 (1.98–3.53)	0.002
Biochemical scores						
Hyaluronic, ng/ml	61.35 (44.43–94.09)	74.98 (46.37–117.55)	0.004	52.18 (40.58–73.89)	84.50 (53.87–140.50)	< 0.001
Cholyglycine, ug/ml	2.65 (1.48–5.26)	2.30 (1.38–5.79)	0.365	1.98 (1.11–4.46)	3.65 (2.03–7.49)	< 0.001
Laminin, ng/ml	19.17 (10.01–30.40)	31.35 (23.48–41.08)	< 0.001	14.96 (8.25–23.47)	25.46 (16.03–38.31)	< 0.001
PIIINP, ng/ml	26.50 (19.06–39.64)	27.45 (21.99–37.71)	0.065	21.10 (16.43–27.57)	38.06 (27.16–53.96)	< 0.001
Type IV collagen, ng/ml	26.11 (19.05–37.80)	25.90 (20.78–36.35)	0.410	21.60 (15.67–26.96)	37.10 (26.77–53.11)	< 0.001
APRI	0.68 (0.38–1.37)	0.75 (0.38–1.83)	0.095	0.49 (0.29–0.94)	0.97 (0.58–1.82)	< 0.001
FIB-4	1.33 (0.92–2.15)	1.32 (0.95–2.26)	0.444	1.17 (0.85–1.66)	1.69 (1.07–3.00)	< 0.001
GPR	0.23 (0.11–0.50)	0.55 (0.26–1.33)	< 0.001	0.15 (0.08–0.29)	0.38 (0.21–0.81)	< 0.001

NAFLD, nonalcoholic fatty liver disease; ALT, alanine transaminase; AST, aspartate aminotransferase; ALP, alkaline phosphatase; GGT, γ-glutamyl transpeptadase; TBil, total bilirubin; DBil, direct bilirubin; FBG, fasting blood glucose; TC, total cholesterol; TG, triglyceride; LDC, low-density lipoprotein; HDL, high-density lipoprotein; INR, international normalized ratio; APTT, activated partial thromboplastin time

### Identification of predictive factors for advanced liver fibrosis and cirrhosis

By using logistic regression analysis, we identified variables related to advanced liver fibrosis and cirrhosis. The presence of advanced liver fibrosis (S3-S4) was associated with AST, ALP, TBil, DBil, cholinesterase, albumin, globulin, prealbumin, total bile acid, TC, TG, LDL, prothrombin time, INR, thrombin time, RBC, platelet count, neutrophil count, hyaluronic acid, cholylglycine, laminin, PIIINP, and type IV collagen. Multivariable analysis identified INR (*P* = 0.003, odds ratio [OR] = 1.58, 95% confidence interval [CI] = 1.17–2.15), platelets (*P* < 0.001, OR = 0.99, 95% CI = 0.98–1.00), and PIIINP (*P* < 0.001, OR = 1.06, 95% CI = 1.04–1.09) as independent predictors of advanced liver fibrosis.

The presence of cirrhosis (S4) was associated with AST, ALP, TBil, DBil, cholinesterase, albumin, globulin, prealbumin, total bile acid, TC, TG, LDL, PT, INR, fibrinogen, thrombin time, WBC, platelet count, neutrophil count, hyaluronic acid, cholylglycine, laminin, PIIINP, and type IV collagen. Multivariable analysis identified INR (*P* = 0.001, OR = 1.85, 95% CI = 1.29–2.15), platelets (*P* < 0.001, OR = 0.97, 95% CI = 0.96–0.98), and PIIINP (*P* < 0.001, OR = 1.02, 95% CI = 1.01–1.04) as independent predictors of cirrhosis.

### Predictive nomogram construction and calibration in the training cohort

**Fig. 1:**
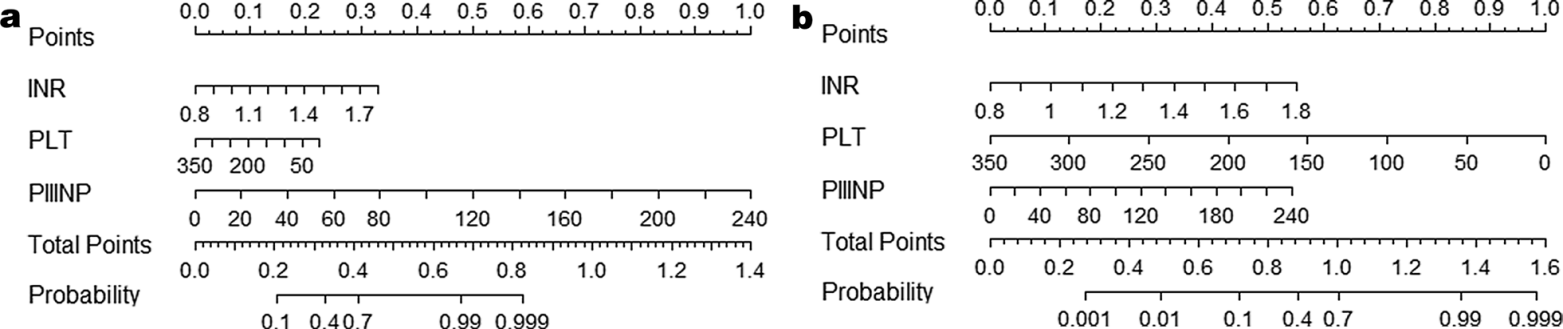
Liver fibrosis Nomograms in training set. The nomogramS3S4 (**a**) and the nomogramS4 (**b**) were constructed for evaluation of advanced fibrosis and cirrhosis, respectively. Each variable is assigned a point on the top axis by drawing a line upward. The sum of these numbers is located on the Total Points axis, and a line is drawn downwards to the Probability axis to identify the likelihood of advanced fibrosis and cirrhosis

The predictive nomograms that combined all significant independent predictive factors for advanced fibrosis and cirrhosis in the training cohort are shown in Fig. 1. The independent factors included platelet counts, INR, and PIIINP. A sum score could be calculated as the total scores of related predictors and referred to the probability of advanced fibrosis or cirrhosis in the basal axis. For example, in a patient whose INR was 1.1, their platelet count was 200 × 10^9^/L, PIIINP was 58 ng/ml, total points scored was 0.42, and advanced fibrosis probability was approximately 70%; in a patient whose INR was 1.1, platelet count was 200 × 10^9^/L, their PIIINP was 140 ng/ml, total points scored was 0.88, and cirrhosis probability was approximately 40%.

**Fig. 2:**
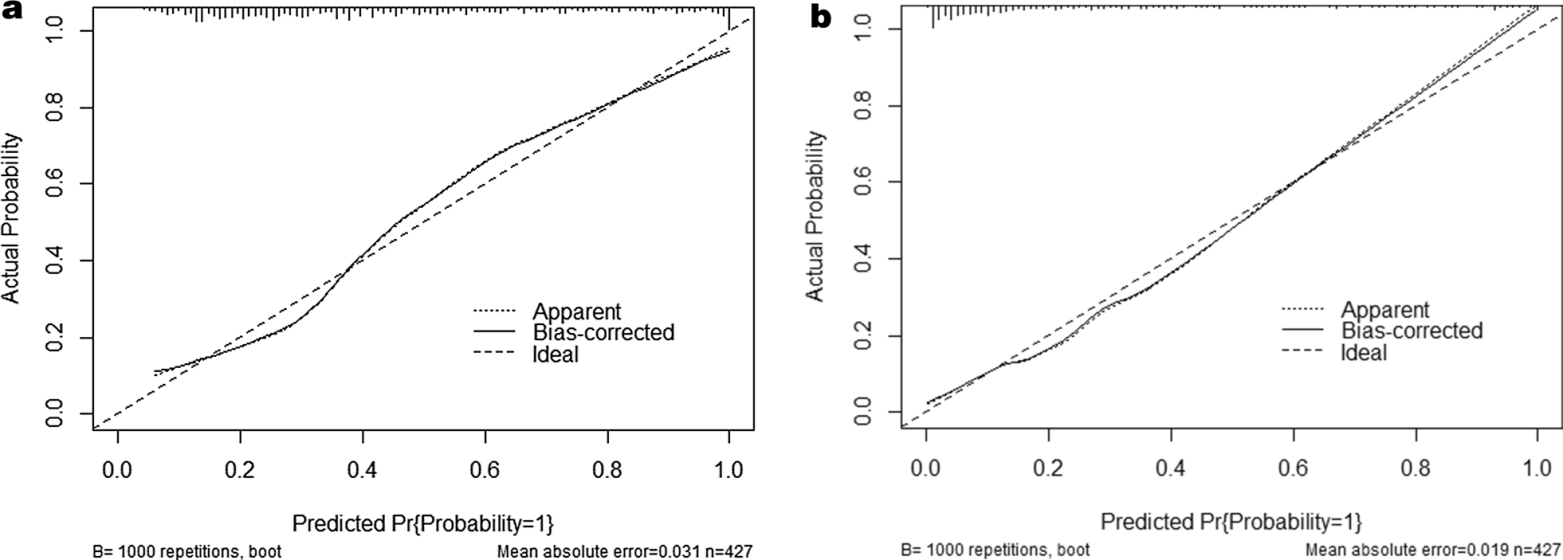
Liver fibrosis calibration curves in training set. The calibration curves for predicting advanced fibrosis (**a**) and cirrhosis (**b**) in chronic liver disease patients. Nomograms-predicted probability of liver fibrosis stages is plotted on the x-axis, and actual probability is plotted on the y-axis

The calibration plot for the probability of advanced fibrosis showed optimal agreement between the prediction by the nomogram S3S4 and the actual observation (Fig. 2a). Remarkably, the calibration plot for the probability of cirrhosis showed good consistency between the nomogram S4 prediction and actual observation (Fig. 2b).

### Comparison of the predictive accuracy between nomograms and noninvasive markers in the training cohort and validation cohort

**Fig. 3:**
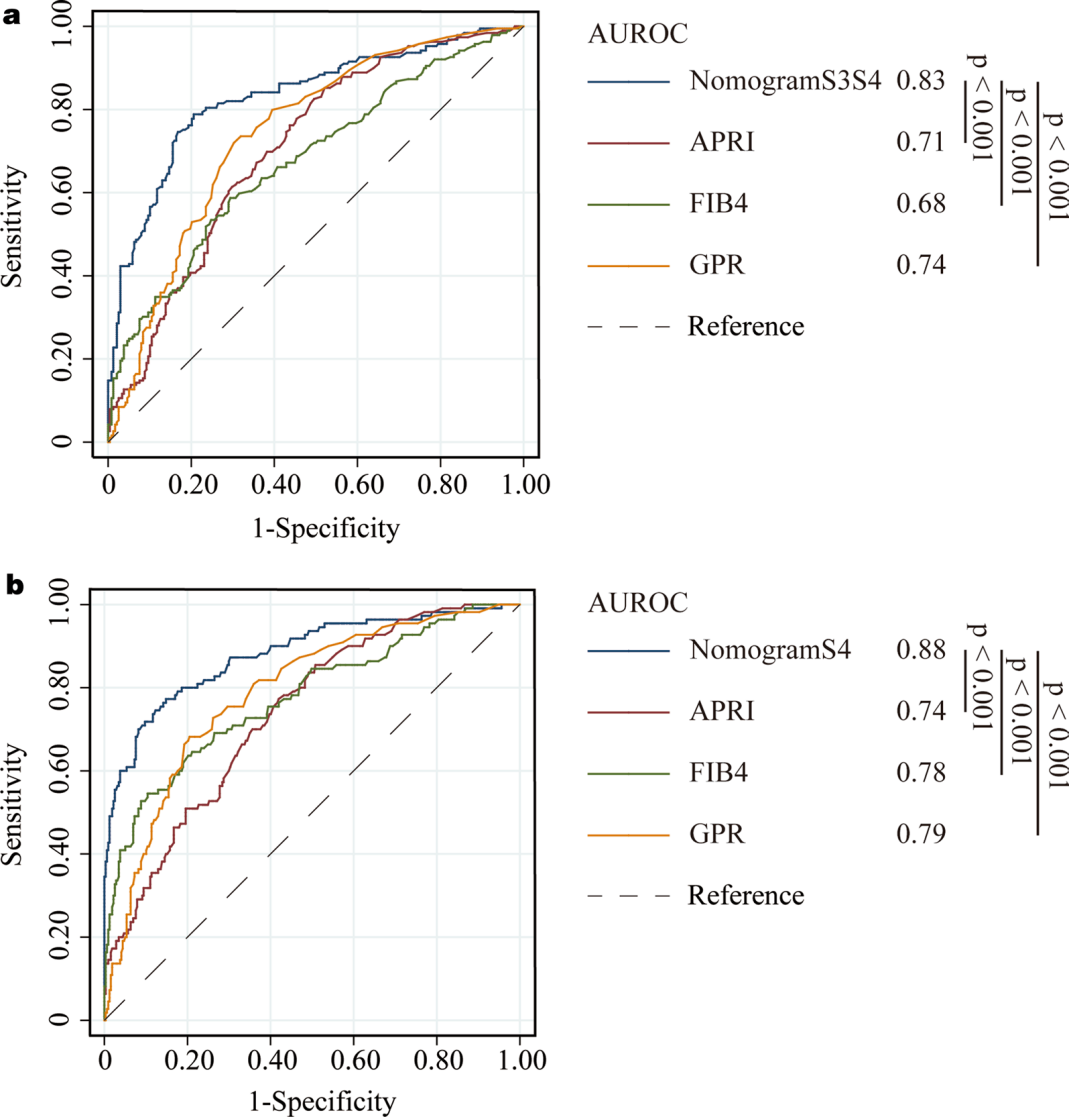
Area under receiver operating characteristic (ROC) comparison of Nomograms, APRI, FIB-4, and GPR in training set. **a** ROC comparison for predicting advanced fibrosis; **b** ROC comparison for predicting cirrhosis

**Fig. 4:**
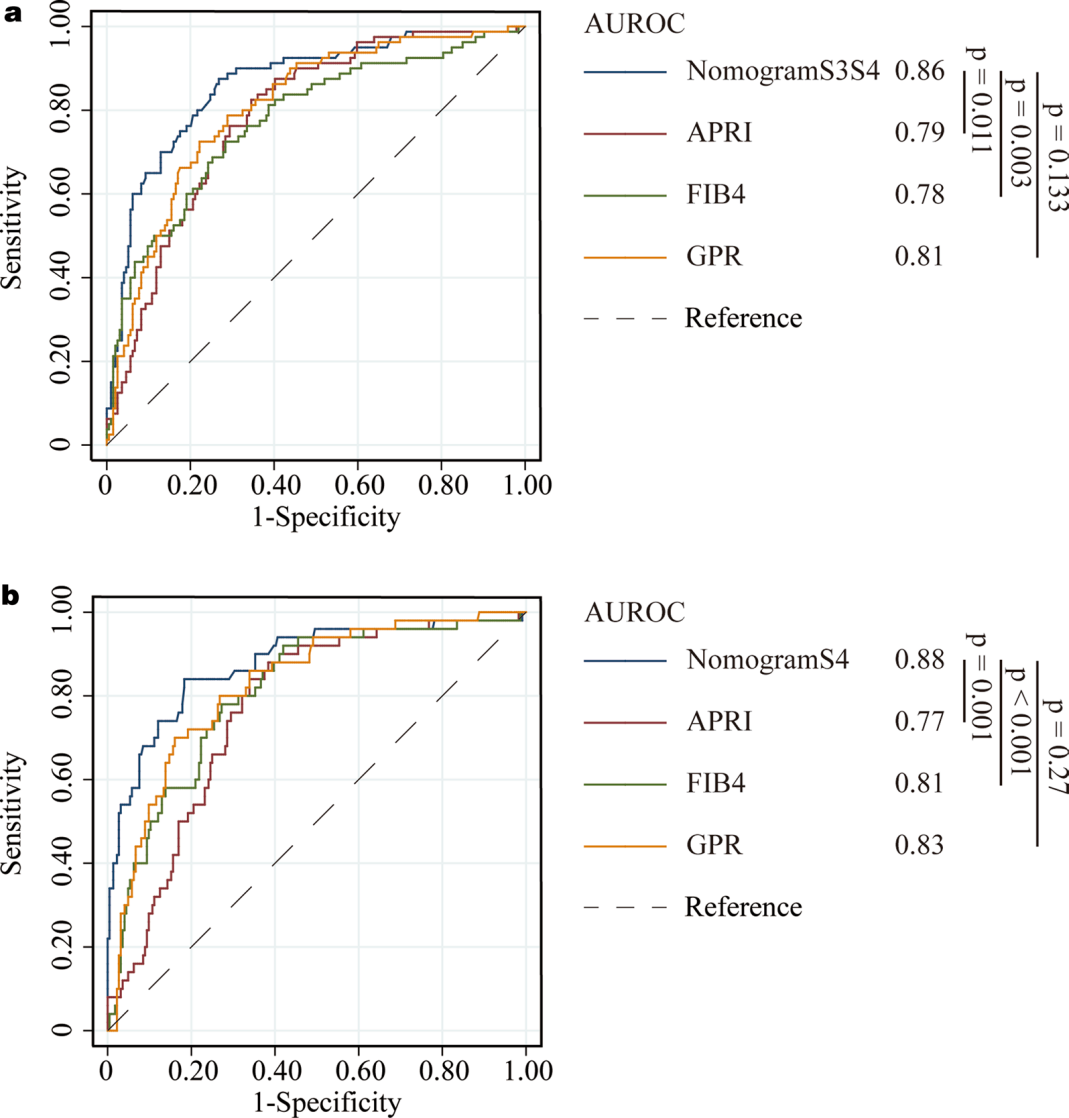
Area under receiver operating characteristic (ROC) comparison of Nomograms, APRI, FIB-4, and GPR in validation set. **a** ROC comparison for predicting advanced fibrosis; **b** ROC comparison for predicting cirrhosis

The established nomograms displayed better accuracy in predicting advanced fibrosis and cirrhosis. The ROC curves for our nomograms, APRI, FIB-4, and GPR are shown in training set (Fig. 3) and validation set (Fig. 4), respectively. In the training set, for discriminating advanced fibrosis, Nomogram S3S4 had the highest areas under the ROC curve (AUROC) (0.83, sensitivity 78.9% and specificity 79.4%) compared with APRI (0.71, sensitivity 81.5% and specificity 51.7%), FIB-4 (0.68, sensitivity 38.6% and specificity 90.6%), and GPR (0.74, sensitivity 73.2% and specificity 69.3%). When discriminating cirrhosis, nomogram S4 had the best AUROC (0.88, sensitivity 77.3% and specificity 85.2%) compared with APRI (0.74, sensitivity 78.2% and specificity 57.8%), FIB-4 (0.78, sensitivity 53.7% and specificity 90.1%), and GPR (0.79, sensitivity 67.3% and specificity 80.8%) (Table 2). Similarly, in the validation set, compared to the other three serum indexes, nomogram S3S4 had the highest AUROC (0.86, sensitivity 70.0% and specificity 87.1%) for predicting advanced fibrosis. nomogram S4 had the best AUROC (0.88, sensitivity 54.0% and specificity 95.9%) for predicting cirrhosis (Table 3). These results suggest that the nomograms were useful predictors of advanced fibrosis and cirrhosis for patients with chronic liver disease.

**Table 2:** Predictive performances of nomograms, APRI, FIB-4, and GPR for advanced fibrosis (S3–S4) and cirrhosis (S4) (Training cohort)

Indexes	AUROC (95%CI)	*P*	Cutoff	Se (%)	Sp (%)
S3–S4					
NomogramS3S4	0.83 (0.79–0.87)	< 0.0001	0.41	78.9	79.4
APRI	0.71 (0.66–0.75)	< 0.0001	0.51	81.5	51.7
FIB-4	0.68 (0.60–0.70)	< 0.0001	1.48	58.7	70.6
GPR	0.74 (0.70–0.79)	< 0.0001	0.23	73.2	69.3
S4					
NomogramS4	0.88 (0.85–0.91)	< 0.0001	0.91	77.3	85.2
APRI	0.74 (0.70–0.79)	< 0.0001	0.66	78.2	57.8
FIB-4	0.78 (0.73–0.81)	< 0.0001	2.34	54.6	89.6
GPR	0.79 (0.75–0.83)	< 0.0001	0.38	67.3	80.8

AUROC, area under ROC; Se, sensitivity; Sp, specificity

**Table 3:** Predictive performances of nomograms, APRI, FIB-4, and GPR for advanced fibrosis (S3-S4) and cirrhosis (S4) (Validation cohort)

Indexes	AUROC (95%CI)	*P*	Cutoff	Se (%)	Sp (%)
S3–S4					
NomogramS3S4	0.86 (0.82–0.90)	< 0.0001	0.41	70.0	87.1
APRI	0.79 (0.73–0.83)	< 0.0001	0.51	91.2	47.0
FIB-4	0.78 (0.72–0.82)	< 0.0001	1.48	72.5	68.6
GPR	0.81 (0.76–0.85)	< 0.0001	0.23	97.5	28.9
S4					
NomogramS4	0.88 (0.83–0.92)	< 0.0001	0.91	54.0	95.9
APRI	0.77 (0.72–0.82)	< 0.0001	0.66	92.0	53.6
FIB-4	0.81 (0.76–0.85)	< 0.0001	2.34	58.0	84.4
GPR	0.83 (0.78–0.87)	< 0.0001	0.38	94.0	46.4

AUROC, area under ROC; Se, sensitivity; Sp, specificity

### DCA for clinical utility of the established nomograms

**Fig. 5:**
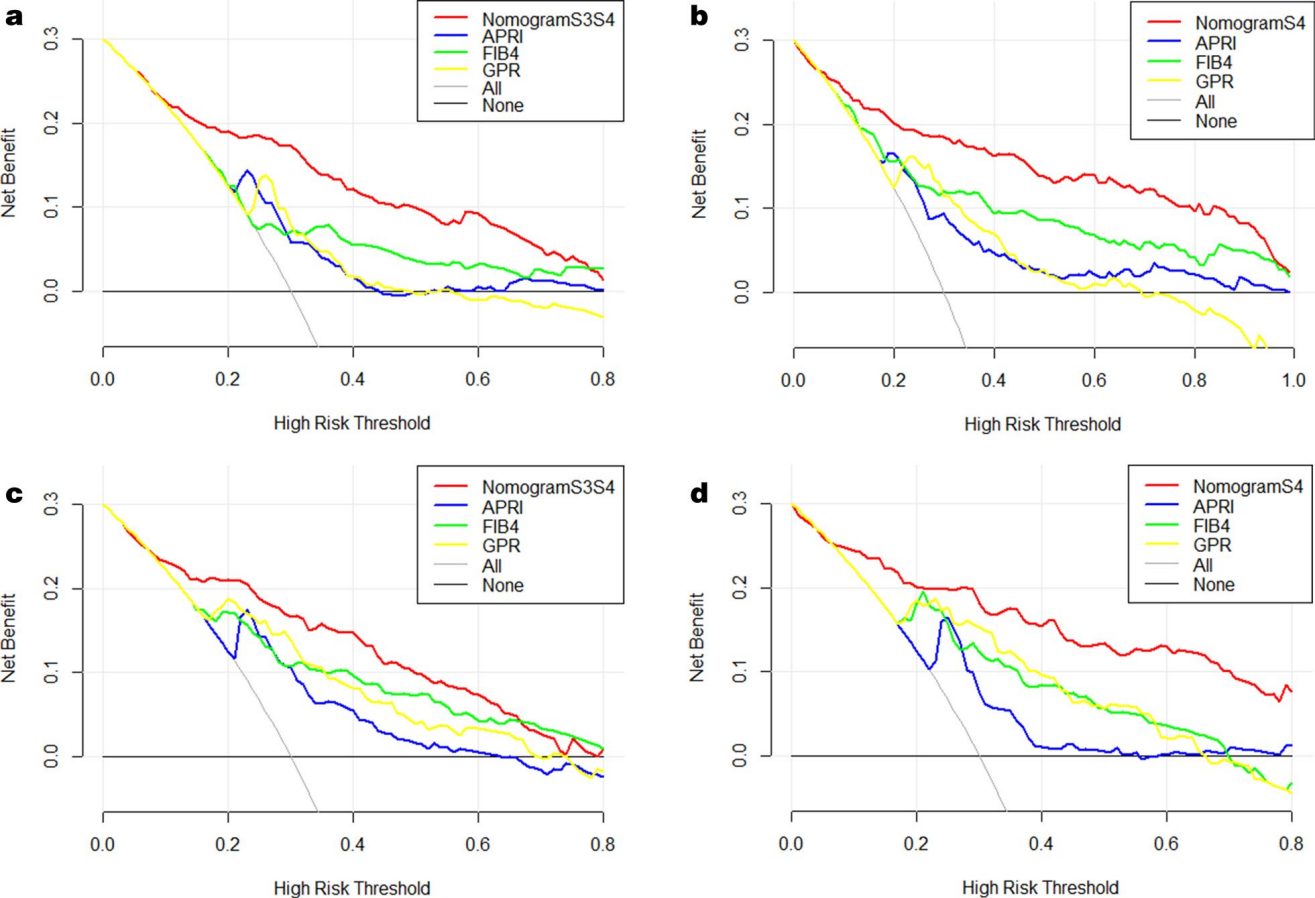
Liver fibrosis decision curve analysis. Decision curve analysis depict the clinical net benefit. NomogramS3S4 is compared with APRI, FIB-4, and GPR for predicting advanced fibrosis in the training set (**a**); NomogramS4 is compared with APRI, FIB-4, and GPR for predicting cirrhosis in the validation set (**b**); NomogramS3S4 is compared with APRI, FIB-4, and GPR for predicting advanced fibrosis in the training set (**c**); NomogramS4 is compared with APRI, FIB-4, and GPR for predicting cirrhosis in the validation set (**d**). Black line = net benefit when no patient will experience the event; gray line = net benefit when all patients will experience the event. The preferred markers is the marker with the highest net benefit at any given threshold

In addition, we conducted DCA to further investigate the clinical application values of the nomograms in predicting advanced fibrosis and cirrhosis. In the training set, DCA revealed that from a threshold probability of 10–80%, the application of nomogram S3S4 to predict advanced fibrosis risk increased the benefit considerably more than the other three scores (Fig. 5a). In particular, employing nomogram S4 allowed us to obtain much more net benefit at any threshold probability (Fig. 5b). As we expected, the DCAs of the validation set and our nomograms also showed a better net benefit with a wide range of threshold probabilities and better performances for predicting advanced fibrosis and cirrhosis than APRI, FIB-4, and GPR (Fig. 5c, d).

## Discussion

Although all international guidelines recommend early treatment for patients with chronic liver disease, it is estimated that two-thirds of patients with cirrhosis are diagnosed in time when they have liver-related complications, compromising their short-term prognosis. In our study, the INR, PLT, and PIIINP were independent risk factors for progressive liver fibrosis in patients with chronic liver disease [22]. This indicates that there is an urgent need for simple and accurate tools to identify the subgroup of chronic liver disease patients who require special treatment by professional physicians. We subsequently constructed noninvasive nomograms that incorporated the three indexes to predict advanced fibrosis and cirrhosis in chronic liver disease patients to assess the progression of liver fibrosis in a noninvasive procedure in chronic liver disease patients. The established nomograms are convenient, useful and user-friendly in clinical practice.

INR is a known parameter often used in the assessment of liver fibrosis. Sterlin et al. [23] reported that the INR was an independent predictor for liver fibrosis and that its level correlated directly with liver function. Cross et al. [24] reported that the INR level correlated with liver fibrosis and was used as a parameter in the Kings score, which was helpful to diagnose liver fibrosis. Furthermore, Takaki et al. [25] reported that the INR correlates independently and significantly with significant liver fibrosis in hepatitis C patients and used it to develop the VIA index to measure liver fibrosis stage.

The process of liver fibrosis is thought to be caused by excessive production of extracellular matrix (ECM). In the liver type III collagen mainly occurs [26]. During the synthesis of type III collagen, PIIINP is detached from procollagen type III [27], so fibrogenesis results in the release of ECM fragments into the blood. Thus, circulating levels of PIIINP can be a biomarker of hepatic fibrogenesis [27, 28]. In some observational studies, plasma PIIINP levels have been shown to be strongly associated with liver fibrosis in patients with chronic liver disease [29–32]. The best predictive value was reported, with an AUROC = 0.87 distinguishing fibrosis and cirrhosis from normal liver or steatosis in 45 cases with alcoholic liver disease [31]. A small study demonstrated that the combination of genetic variations and enhanced liver fibrosis (ELF) indexes (tissue inhibitor of matrix metalloproteinase-1, hyaluronic acid, and PIIINP) could predict the progression of fibrosis in 56 patients with mid-liver fibrosis over 5 years [33]. Another study showed that compared with other clinical scores, the ELF test for 457 HCV patients could better predict morbidity and mortality after 7 years of follow-up [34]. Nielsen et al. [35] developed the PRO-C3 assay (a neoepitope-specific serum ELISA for type III procollagen) that could accurately predict advanced fibrosis in patients with nonalcoholic fatty liver disease (NAFLD). Recently, a PRO-C3-based fibrosis algorithm that included age, diabetes, PRO-C3, and platelet count (ADAPT) was developed to more accurately identify advanced fibrosis in patients with NAFLD compared to APRI, FIB-4, and NAFLD fibrosis score [36]. One possible explanation for the increased PIIINP level in advanced fibrosis is that ECM remodeling is an attempt to repair damaged tissue in response to liver injury. If liver damage is extensive, ECM components and collagen synthesis will be increased.

Several new noninvasive methods for the prediction and assessment of liver fibrosis have recently become available. In this study, the predictive power of the established nomograms in the assessment of liver fibrosis was excellent in both the training group and validation group and was significantly higher than that of the APRI, FIB-4, and GPR. The nomograms provided better sensitivity and specificity than the other three markers for staging advanced fibrosis and cirrhosis. Moreover, in the DCA analysis, a new analysis that visually predicted the clinical applicability of a model, our nomograms demonstrated a superior benefit across a wider range of threshold probabilities compared with APRI, FIB-4, and GPR.

APRI and FIB-4 are the two widely studied noninvasive methods for assessing liver fibrosis stage. Teshale et al. [37] assessed the predictive values of APRI and FIB-4 in a large cohort of chronic hepatitis B patients and demonstrated that APRI and FIB-4 distinguished significant liver fibrosis with good sensitivity and specificity. The WHO hepatitis B guidelines have recommended APRI to predict liver fibrosis stage in resource-limited countries [12]. In a meta-analysis pooling 4266 HCV patients from 22 studies, the AUROCs of ARPI for diagnosing significant fibrosis and cirrhosis were 0.76 and 0.86, respectively [38]. However, another meta-analysis indicated that APRI and FIB-4 had only moderate sensitivity and accuracy for diagnosis of liver fibrosis in chronic hepatitis B patients, which suggested they were not ideal substitutes for liver biopsy [39]. Regarding NAFLD, recent studies showed that APRI could better distinguish fibrosis stage F2-3 from F0-1 than FIB-4 [40, 41]. The GPR developed by Lemoine et al. was shown to be more accurate than the APRI and FIB-4 [42]. Additionally, GPR is reported to predict significant fibrosis and cirrhosis excellently in a large cohort of chronic hepatitis B Gambian patients using FibroScan measures as a reference [43]. However, it did show a controversial advantage in several Chinese cohorts [8, 44].

Our study has several limitations. First, this is a retrospective study in a single center, so it should be validated with more patients from other centers. Second, further prospective studies are needed to investigate whether the established nomograms had disease-specific cutoffs to distinguish stages of liver fibrosis.

## Conclusion

In summary, these results suggest that serum INR, PLT, and PIIINP are independent predictors of advanced liver fibrosis and cirrhosis. Furthermore, the nomogram S3-4 and nomogram S4 established based on the three parameters demonstrate superior clinical usefulness. Although future validation in a large cohort is needed, these established nomograms in a noninvasive manner could be promising procedures for staging liver fibrosis, especially in resource-limited regions.

## Data Availability

Datasets of the current study are available from the corresponding authors on reasonable request.

## Abbreviations

APRI: Aspartate transaminase to platelet ratio
FIB-4: Fibrosis-4 score
GPR: γ-Glutamyl transpeptidase to platelet ratio
INR: International normalized ration
ALT: Alanine transaminase
AST: Aspartate aminotransferase
ALP: Alkaline phosphatase
GGT: γ-Glutamyl transfetase
PIIINP: N-terminal propeptide of type III procollagen
ULN: Upper limit of normal
ROC: Receiver operating characteristics
DCA: Decision curve analysis
AUROC: Areas under ROC
ECM: Extracellular matrix
ELF: Enhanced liver fibrosis indexes
NAFLD: Nonalcoholic fatty liver disease
